# Therapeutic Notes

**Published:** 1896-12-19

**Authors:** 


### THERAPEUTIC NOTES.

### FERRALIN.

Ferralin is one of the many new organic preparations of iron, which is reported on the Continent to be most useful in the treatment of anæmic conditions. It is a reddish brown, tasteless, and inodorous powder. It was prepared in the first instance by Schunedeburg and his pupil Marfori, from the liver of pigs. They subsequently discovered that a similar body could be made artificially from white of egg, treated in an alkaline medium with an iron salt. In the chemical analysis, to which it was subsequently submitted by Marfori (*Ann. di Chimica e di Farmacol.*, Feb., 1894), it was found that iron existed to the extent of 7 to 8 per cent. Experiment showed that it was readily absorbed from the intestinal tract, and that when injected direct into veins it disappeared from the blood, but was not readily excreted by the kidneys or intestines. These observations find confirmation in the later researches of De Filippi (*Zeigl. Beitrag. Bd. xvi.*, p. 462), for he has experimentally shown that when absorbed by subcutaneous injection Ferralin finds its way in large quantities into the bone marrow, and its presence then may be demonstrated by microchemical tests. The peripheral lymph glands appear also to contain traces of this substance, as also do leucocytes in other situations. The kidneys, however, appear to show little or no evidence that it is being excreted by their agency. If Ferralin was injected by the intravenous method De Filippi discovered the presence of its iron in the spleen, liver, bone marrow, and in the lungs. But in this connection it must be remembered that the liver of many animals contains a substance closely allied to Ferralin, and that many other substances, such as hæmalogen, may easily be confounded with it. But it seems practically certain that in whatever way Ferralin is introduced into the system, its passage through the body can be followed by the evidence of leucocytes loaded with iron. Clinically, Ferralin is well spoken of by Von Ziemssen, Kundig, and other Continental observers. Not only is the number of the red blood corpuscles increased by its administration, but their hæmoglobin contents is increased, and the general condition of the patient is improved. The dose is from 8 to 15 grains, two or three times a day.

